# Set of stress biomarkers as a practical tool in the assessment of multistress effect using honeybees from urban and rural areas as a model organism: a pilot study

**DOI:** 10.1007/s11356-020-11338-2

**Published:** 2020-10-30

**Authors:** Łukasz Nicewicz, Agata W. Nicewicz, Alina Kafel, Mirosław Nakonieczny

**Affiliations:** Research Team of Animal Physiology and Ecotoxicology, Faculty of Natural Sciences, Institute of Biology, Biotechnology and Environmental Protection, University of Silesia, Bankowa 9, 40-007 Katowice, PL Poland

**Keywords:** Biomarkers, Detoxification, Environmental stress, Enzyme activity, Honey bee, Laboratory tests, Urban beekeeping

## Abstract

A decrease among honey bee populations (*Apis mellifera*) in the traditional apiaries has been observed in recent years. In light of this negative phenomenon, urban beekeeping seems to be an appropriate alternative solution for the bee population in reducing the toxic effects of a large number of pesticides that are commonly used in agricultural ecosystems. Despite the rapid development of urban beekeeping, there is little information regarding the different aspects of the defense effectiveness of bees from the urban and rural areas. The study was aimed to show whether honey bees from these two locations differ in the level of the valuable biomarkers of stress exposure helpful in establishing which bees, from urban or rural areas, are under greater environmental pressure. For this purpose, foragers from an urban rooftop apiary and a traditional rural apiary were collected. The chosen biomarkers were measured in various tissues of bees. The activity of glutathione *S*-transferase and acetylcholinesterase, the level of total antioxidant capacity, heat shock protein 70 (Hsp70), and defensin were selected for the analyses. In our opinion, the Hsp70 and defensin levels seemed to be important in the indication of urban multistress factors. The higher level of heat shock proteins and defensins in tissues/organs of bees from the urban apiary—in the gut (an increase, respectively, 92% and 7.3%) and fat body (an increase, respectively, 130% and 7.8%), known as targets of environmental toxins, pointed out the urban environment as highly stressful at both the individual and colony levels. In turn, high total antioxidant capacity was measured in the guts of honey bees from rural area (an increase 107%). Such a situation suggests a different mechanism of defense and specificity of rural and urban environmental stressors and also honey bees foraging activity.

## Introduction

In recent years, there has been noted a decrease in the honey bee populations, especially in Europe (Potts et al. 2010) and North America (Ellis et al. 2010). In the USA, annual losses of bee colonies in 2011–2016 were estimated at as much as 50% (van Engelsdorp et al. 2017). An increased intensification of agriculture, especially the use of plant protection chemicals and the loss of habitat diversity, has affected bees significantly, both at the individual and colony levels (van Engelsdorp and Meixner 2010; Johnson et al. 2010). Additionally, pesticides alter the susceptibility of bees to pathogens and may promote the spread of diseases (Pettis et al. 2012). However, the reasons for the decrease in honey bee populations may be multiple and can include not only unfavorable changes, diseases, and viruses, but also an insufficient quantity and quality of pollen due to the intensive agricultural practices (Maxim and Van Der Sluijs 2012). The loss of honey bee populations has caused an increase in the urban beekeeping interest, most notably in agglomerations (Lorenz and Kerstin 2015). Urban environments can be characterized by significantly lower use of pesticides, but also a higher content of heavy metals, air pollution, greater food base fragmentation, vehicle traffic, and more complicated spatial structures, which may all affect the welfare of bees (Cariveau and Winfree 2015; Negri et al. 2015; Rachael et al. 2009; Sadowska et al. 2019). All those environmental features are distinguished by a long term of acting on organisms on the NOAEL level and they are considered as a multistress (van Straalen 2003).

Despite the rapid development of urban beekeeping, there is scarce information about the condition of bees from beehives that are located in cities. There are several publications on the content of heavy metals in bees and in bee products such as honey or wax (Conti and Botrè 2001; Giglio et al. 2017; Lambert et al. 2012; Leita et al. 1996; Sadowska et al. 2019). The report of Lecocq and co-authors indirectly analyzed only the conditions of bees using their productivity, and therefore, there is still a need to know how the stress factors specific for urban environments may affect the welfare of bees (Lecocq et al. 2015).

Honey bee foragers are exposed to contaminants present in the air, water, soil, and vegetation that may be brought back to the hive and spread among the entire colony (Negri et al. 2015). The question is do an urban environment that is characterized by multiple urban stressors affect bees at both the individual and colony levels?

The chosen valuable stress exposure biomarkers are presented as sensitive to different environmental stressors, so the aim of the study was to compare biochemical endangerment in bees from the urban and rural apiary. To be specific, the biomarkers were measured in different tissues, among the others typical targets of environmental toxins, gut and fat body of the foragers, and in tissues which were connected with the exposure of toxins characterized high retention rate within the insects bodies, muscles or brain. It could be helpful in the assessment of specificity of urban and rural environmental pressure.

For this aim, well-known biomarkers were analyzed in various tissues of the bees: glutathione *S*-transferase (GST), acetylcholinesterase (AChE), total antioxidant capacity (TAC), heat shock protein 70 (Hsp70), and defensin level. GSTs are widely used as biomarkers of exposure to oxidative stress in invertebrates (Yan et al. 2013). The AChE activity may indicate that an animal was exposed to organophosphorus or carbamate insecticides (Nauen et al. 2001). GST and AChE response to insecticides was presented under exposure of four insecticides in individuals of *A. mellifera* (Badawy et al. 2015) and heavy metals (Nikolić et al. 2019). New data has indicated that AChE may be involved in the manifold stress response or stress management (Kim et al. 2017). TAC is another parameter measuring the almost complete antioxidative status of animals that are exposed to stress concerning their general “crude” antioxidant response level (Johnson and Carey 2014; Słowińska et al. 2016). Hsp70 is routinely used in ecosystem biomonitoring (Köhler et al. 1996). A high level of the Hsp70 indicates that an animal is under stress that exceeds the threshold for the damage to cells to be repaired and also affects the detoxification processes (Koban et al. 1991). Defensin is an antimicrobial peptide that is involved in the humoral immunity in honey bees together with apidaecin, abaecin, and hymenoptaecin. Its increased level may indicate animal life under stress factors that can reduce immunity (e.g., Antúnez et al. 2009; Casteels-Josson et al. 1994; Rand et al. 2015). Johnson (2015) emphasized “The honey bee colony is a nexus for all of the toxic compounds that exist in the environment” or that honeybees may be especially sensitive to certain insecticides and that their sensitiveness is moderated through many biotic or abiotic circumstances. The examination of the honey bees foraging in variably stressed environments as bioindicator is highly valuable, referring to, among the others, their immune or antioxidant response (Li et al. 2018).

We hope that our results will be helpful in the achievement of better legitimacy for the introduction of beekeeping into cities.

## Material and methods

### Honey bees

All of the experiments were carried out on European-derived Carniolan forager bees (*Apis mellifera carnica* Pollman, 1879). The insects were collected from two apiaries located in urban and rural areas. These areas are described by the new degree classification of urbanization defined according to the population size, density, and contiguity of local administrative units level 2 (LAU2) by the European Commission (Dijkstra and Poelman 2014; Eurostat 2016). A conventional apiary is located in a rural area: 52° 18′ 08.2″ N, 22° 08′ 59.9″ E, and urban apiary is located in the center of the city—Katowice: 50° 15′ 51.0″ N, 19° 01′ 12.3 ″ E. The first apiary consists of 60 hives that are situated in a typical polish agricultural landscape and it is 20 years old. The urban apiary consists of five hives located on the rooftop of a 30-m high building in the center of Katowice city. This apiary has been run in this place for 3 years.

Even though both apiaries are located in different (south and north) parts of Poland, they are characterized by comparable weather condition components: temperature, atmospheric pressure, wind, humidity, precipitation, and cloudiness (average-year and chosen month data) (Table 1). Whereas, according to data obtained from two urban environmental pollution monitoring stations (the type of station, background level of pollution) located in Katowice and Siedlce (30 km from rural apiary locality) indicated from 2 to 4 times higher concentration of suspended dust (PM10 and PM2.5) or measured heavy metals, arsenic, and benzo-α-pyrene concentrations (Table 2). Katowice and the neighboring area are known as Silesia Agglomeration and it is recognized as a heavily contaminated place in Central Europe for years (Leśniok 2011), while the countryside of North-Eastern Poland is perceived as a “green lungs” of Europe (Szeszko 2017). The plants which are a potential food base for bees from both apiaries are growing in totally different circumstances—post-industrial and urban areas versus extensive agriculture areas.

**Table 1:** Average year and June temperature and precipitation for each apiary localization in 2017. For the rural apiary, data was used for the nearest weather station in the city of Siedlce. Data source: World Meteorological Organization (WMO) (http://meteomanz.com/) and the Institute of Meteorology and Water Management—National Research Institute (http://meteomodel.pl)

	Weather station			
	Katowice	Siedlce	Katowice	Siedlce
Period of measurement	2017–year		2017–June	
T [°C]	9.2	8.6	18.3	17.4
T. max ave. [°C]	13.6	12.6	24.6	23.1
T. min ave. [°C]	4.8	4.7	12.0	11.6
T, max abs. [°C]	34.3	33.7	33.1	29.6
T, min abs. [°C]	-26.0	-21.5	5.7	5.7
Prec. [mm]	716.7	669.9	34.1	59.9
Prec. days 0.1 mm	178	171	12	12
Days of storm	31	31	4	7
Days of frost	94	80	0	0
Days of snow	57	60	0	0
W. sp. [km/h]	2.6	3.0	2.6	3.0
Rel. h., ave. [%]	76.5	79.8	62.2	68.6
SLP [HPa]	1017.4	1015.8	1014.2	1015.4
Insolation [h]	1713.0	1738.9	291.4	277.4
Cloudy	5.6	5.7	4.8	4.9

*T* temperature, *T. max ave*. average maximum temperature, *T. min ave*. average minimum temperature, *T. max abs*. absolute maximum temperature, *T. min abs*. absolute minimum temperature, *Prec*. precipitation, *Prec. days 0.1 mm* days of precipitation equal to or greater than 0.1 mm, *W. sp*. wind speed, *Rel. h*. air relative humidity, *SLP* sea level pressure, *Insolation* the total time (in hours) during that the sun rays fall directly on the Earth surface, *Cloudy* the degree of sky coverage by clouds on the octane scale (0–8)

**Table 2:** The suspended dust concentration (PM10 and PM2.5) and pollution level of selected pollutants in atmospheric particles for each apiary localization. Data for 2017. Data source: Chief Inspectorate of Environmental Protection in Poland n.d. (http://www.gios.gov.pl/en/)

Index	Averaging time	Katowice			Siedlce*		
		Average	Min	Max	Average	Min	Max
PM10 [μg/m^3^]	1 h	41.643	3.949	671.591	29.038	4.006	360.277
	24 h	41.137	9.800	381.400	29.202	4.720	136.610
PM2.5 [μg/m^3^]	1 h	32.551	3.530	512.891	22.981	2.980	328.689
Pb (PM10) [μg/m^3^]	24 h	0.024	0.003	0.082	0.007	0.001	0.023
As (PM10) [ng/m^3^]		1.731	0.500	5.050	0.629	0.025	3.369
Cd (PM10) [ng/m^3^]		0.993	0.100	9.270	0.241	0.025	0.705
Ni (PM10) [ng/m^3^]		1.571	0.500	5.070	0.750	0.500	7.211
BaP (PM10) [ng/m^3^]		7.306	0.200	32.220	3.013	0.036	15.069

*For the rural apiary, data was used for the nearest Regional Air Pollution Monitoring Station in Siedlce

Inseminated honey bee queens from both apiaries were genetically related—they came from the same line-breeding from the one Bee breeder (“Mellifera apiary”). The honey bee foragers were randomly collected in August 2017 from the entrance to the colony as they return from foraging (Scheiner et al. 2013). The collection of bees was carried out in all hives from the urban apiary and in five selected hives from the rural apiary. To minimalize the effect of the colony on obtained data, the colony strength (number of combs, queen age, number of comb with brood, honey, and pollen) of the five hives from the rural area was comparable to that of the honeybee colonies from the urban apiary. Insects were gathered to plastic vials (one bee per vial) and then transferred to liquid nitrogen. The animals were kept at - 70 °C until the sample preparation.

### Sample preparation

Animals were kept on ice and their central nervous system (brain), fat body, thorax muscles, and gut were dissected at 4 °C according to Carreck et al. (2013). Brains for AChE activity analysis (one brain per sample) were homogenized in 50 μl of 100 mM phosphate-buffered saline (PBS; pH 7.4) (BioShop) with 0.5 μl of Triton X-100 (BioRad). GST activity and TAC were measured in the brain, fat body, and gut homogenized in 20 μl of PBS (pH 7.4). Similarly, the dissected brain, fat body, thorax muscle, and gut for the measurement of the Hsp70 and defensin level were homogenized in 20 μl of 1 M PBS (pH 7.4). For these analysis, one brain, one gut, thorax muscle from one bee, and fat bodies from three bees per each sample were used.

**Fig. 1:**
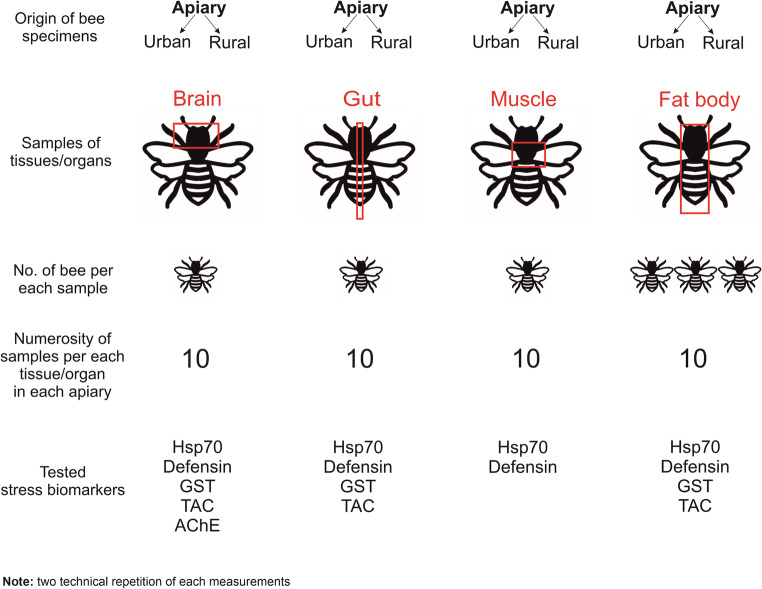
Details of the samples, their repetition, and marking in the study. The number of bees used to prepare one sample was different and depended on the size of the tissues/organs. A total of ten samples were prepared for each tissue/organ and the two technical replications were performed from each of them

Each tissue/organ obtained from bees from both the urban and the rural apiary constituted an experimental group. Tissues/organs of bees from rural apiary were considered as reference (control) groups. In total, eight test groups were distinguished. The experimental and control groups differed from one another in the tested parameters. The number of replicates for the given parameter in each experimental group was ten in two technical repetitions for each. Details about differences between these groups are presented in the Fig. 1.

All homogenates were centrifuged at 4 °C (for acetylocholine esterase (AChE) activity, 12 092*g* for 15 min; glutathione transferase (GST) activity and total antioxidant capacity (TAC), 15 000*g* for 10 min; Hsp70 and defensin level, 3000*g* for 10 min). The supernatants were decanted and the aliquots were kept in Eppendorf microtubes at - 70 °C until the measurements were performed.

The fresh mass of dissected tissues was determined on a laboratory analytical balance before homogenization. The measurements were performed to the scale reading uncertainty ± 0.0001 g.

The total protein concentration in all of the samples was determined according to Bradford (1976).

### Stress biomarkers

The acetylcholinesterase (AChE) activity was analyzed according to the modified method of Ellman et al. (1961) toward acetylcholine iodide as a substrate and expressed in μmoles of hydrolase acetylcholine iodide min^-1^ mg^-1^ total protein.

The glutathione *S*-transferase (GST) activity was measured using a 15-mM ethanol solution of 1-chloro-2,4-dinitrobenzene (CDNB) and expressed in μmoles of the GSH conjugates min^-1^ mg^-1^ total protein (Yu 1996).

The concentrations of heat shock protein 70 and defensin were measured using indirect ELISA, which was performed according to the standard protocol (Crowther 2009) and was optimized according to Pyza et al. (1997) and Chavez-Crooker et al. (2003). Primary anti-Hsp antibody (Mouse Anti-Heat Shock Protein 70 monoclonal antibody, Sigma-Aldrich; 1:1000), anti-defensin antibody (Mouse Anti-defensin monoclonal antibody, Abcam, 1:1000), and the goat anti-mouse IgG Polyclonal Antibody, AP-conjugate (Stressgen) were used. The concentration of Hsp70 was then expressed as the percentage [%] of the total protein and defensin as the absorbance level at 405 nm.

The total antioxidant capacity (TAC) was analyzed according to a modified method of Re et al. (1999) by Kafel et al. (2012). The result was expressed in terms of the equivalent anti-oxidant capacity to the Trolox and expressed as the μmol of the Trolox mg^-1^ protein based on a standard curve.

### Quality assurance and quality control

In the research, we ensured the greatest possible accuracy and precision and the lowest possible uncertainty of the analyses and measurements performed. To reduce systematic uncertainty, (i) all measurements were made by the same researchers, (ii) all measurements (for tissues from bees collected from the urban and rural apiaries) were made at the same time, and (iii) the measurements were started after calibration of the apparatus (if the calibration was possible). Random uncertainty was reduced by performing ten biological repetitions for each sample from tested tissue/organs and two technical repetitions for each sample. The method of sample preparation for each tissue/organ, the numbering of the samples, marking technical, and biological repetitions is presented in Fig. 1.

The uncertainties were quantified. All analyzed parameters were expressed as a mean value from technical and biological repetitions ± average deviation. Scale reading uncertainty was also calculated for the using equipment: the laboratory balance (± 0.0001) and the Tecan Infinite M200 Microplate reader (± 0.0001).

Lack of positive controls for the tested biomarkers in honey bees (Hsp70 and defensin) or the chosen methodology of testing parameters (e.g., AChE and GST activity expressed as a continuous change in the absorbance) is the main problem. The standard curve was used to calculate concentration (total protein) and antioxidant capacity (TAC). The bovine serum albumin (BSA) series solution as a protein standard to the curve creation in the range of 0–1000 ug/ml was used. Series of the Trolox solutions in the range of 0–20 μmol was used to express the antioxidant capacity of tested tissues/organs. The linear calibration and the coefficient of determination (r^2^) were analyzed. A value of r^2^ greater than 0.99 was considered satisfactory. The determination of Hsp70 and defensin levels were based on the total protein concentration based on the standard curve.

The precision of the measurements was analyzed on the basis of the results obtained for the technical repetitions of each sample.

### Statistical analyses

All assays for the statistical analyses were based on ten samples, which were performed in duplicate. The results are reported as the mean values ± SD. Normality was checked using the Kolmogorov-Smirnov test. The significant differences were checked and the Student’s *t* test was used. Results with *p* ≤ 0.05 were considered to be significant. The data were analyzed using GraphPad Prism® ver. 6.

## Results

**Fig. 2:**
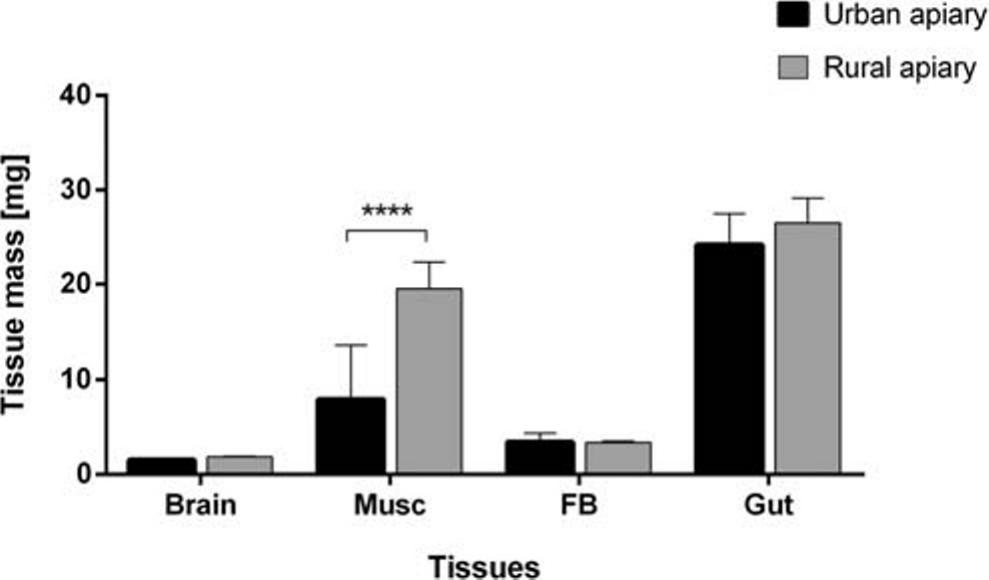
Fresh tissue mass [mean ± SD] obtained from honey bee foragers. Tissue/organ masses are not statistically different in bees from the urban and rural apiary. Musc muscles from the thorax, FB fat body, Gut foregut, midgut, and hindgut. Asterisks indicate statistically significant differences between the same tissues in the bees from the urban and rural apiaries (Student’s *t* test, *p* ≤ 0.05): **** *p* ≤ 0.0001; *N* = 10

The fresh weight of the prepared tissues of central nervous systems, fat bodies, and guts of the honey bees from the urban and rural apiaries was comparable. However, the thorax muscle mass was statistically significantly higher in the bees from the rural apiary (2.5-fold) (Fig. 2).

**Fig. 3:**
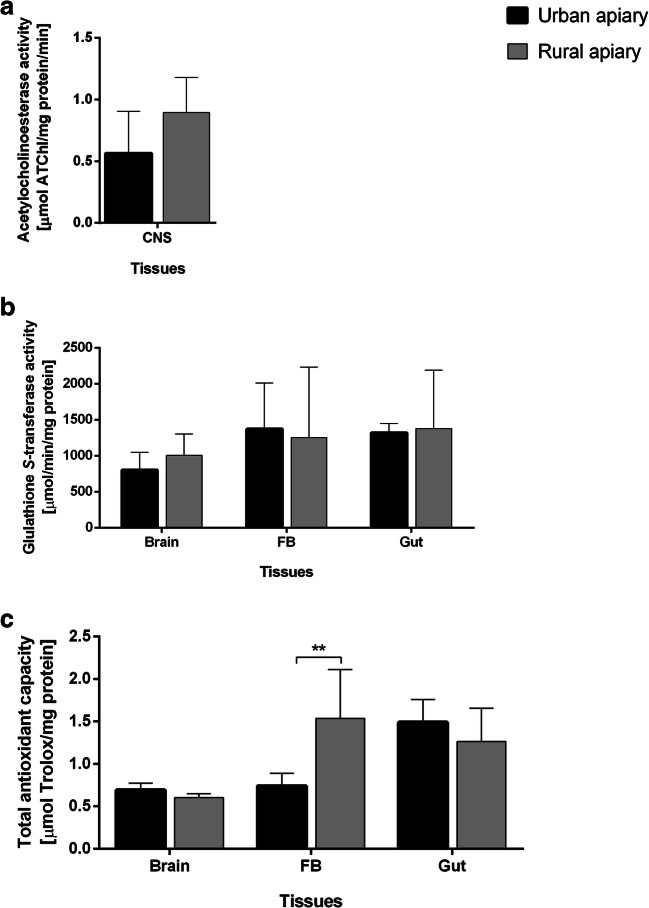
The activity of acetylcholinesterase (AChE) and glutathione *S-*transferase (GST) as well as the level of total antioxidant capacity (TAC) index in examined tissues of honey bee foragers. **a** AChE activity [mean ± SD], **b** GST activity [mean ± SD], and **c** TAC [mean ± SD] are not an appropriate biomarker of exposure to the stress of bees. Musc muscles from the thorax, FB fat body, Gut foregut, midgut, and hindgut. Asterisks indicate statistically significant differences between the same tissues in the bees from the urban and rural apiaries (Student’s *t* test, *p* ≤ 0.05): ** *p* ≤ 0.01; *N* = 10

A higher AChE activity in the honey bees’ origin from rural apiary was statistically insignificant (Fig. 3a).

No variation in total antioxidant capacity was found, when compared results from the brain and gut tissues of honeybees from rural and urban areas. The statistically significant difference in the total antioxidant capacity was observed only in the case of the fat body. It was 2.36-fold higher in the fat body of honey bees from the rural apiary than in those from the urban apiary (Fig. 3c).

**Fig. 4:**
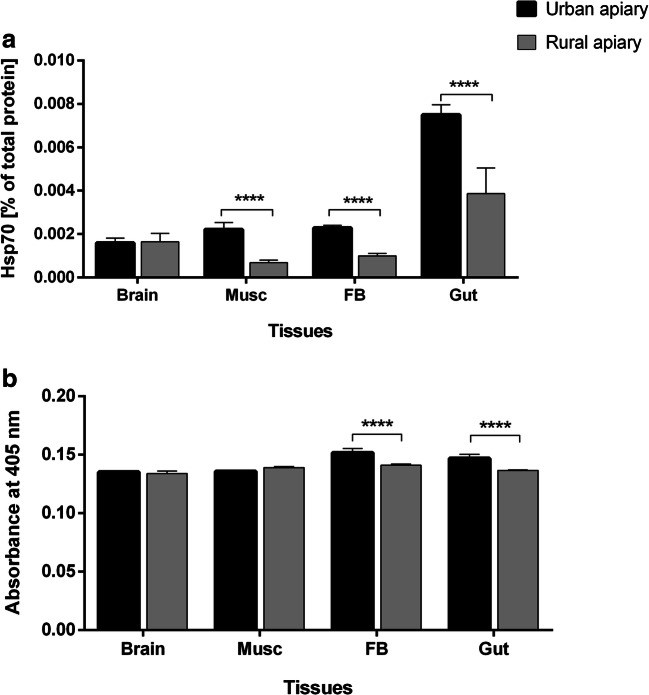
The heat shock protein 70 (Hsp70) and defensin levels in the examined tissues of the honey bee foragers. **a** Hsp70 as a percentage of total protein [mean ± SD] and **b** defensin levels as the absorbance [mean ± SD] seem to be important in the indication of urban multistress factors. brain, Musc muscles from the thorax, FB fat body, Gut foregut, midgut, and hindgut. Asterisks indicate statistically significant differences between the same tissues in the bees from the urban and rural apiaries (Student’s *t* test, *p* ≤ 0.05): **** *p* ≤ 0.0001; *N* = 10

The Hsp70 level in the muscles of the thorax, fat bodies, and guts of the honey bees from the urban apiary was significantly higher (2.85, 2.3, and 1.88-fold, respectively) than in the bees that had been collected from the rural apiary. In turn, a similar level of Hsp70 proteins was presented in the case of honey bees’ brain examination (Fig. 4a).

The defensin level was different in the fat body and gut of the honey bees tissues from tested apiaries and was 1.1-fold higher in the insects from the urban apiary. Brain and muscles of honey bees from both apiaries (rural and urban) did not vary (Fig. 4b).

## Discussion

The localization of the apiary in Katowice was described as an urban area according to the definition of the European Commission (Dijkstra and Poelman 2014; Eurostat 2016). The urban area (alternative name, urban center) is characterized as a densely populated area (at least 50% population living in high-density clusters). Whereas, the rural apiary localization was defined as a thinly populated area (alternative name, rural area) where more than 50% of the population living in rural grid cells (Dijkstra and Poelman 2014). Urban environments are characterized by permanent higher exposure to potential multistress factors, such as the pollutions that are emitted by vehicle traffic, heating, and processing industries or burning of unappropriated fuels. Furthermore, the phenomenon of heat islands, the spatial complexity of the center of cities, and the fragmentation of the food base can be observed in cities too (Lowenstein et al. 2014; Solecki et al. 2005). Therefore, the specificity of the stressors that act on the honey bees from urban apiaries (frequently on the NOEL level) compared to their situation in agricultural apiaries should be emphasized. The concentration of pesticides and heavy metals and organic compounds pollution were not measured in this research. However, the data obtained from GIOŚ for the apiary localization (Table 2) and literature data (Table 3) encourages to the comparative analysis of the level of environmental pressure on bees in these two different habitats. The rural apiary is situated in a typical polish agricultural landscape with low-area crops maize, rape or buckwheat, pesticide use (inefficient and excessive, Nowak and Bury 2017), and temporary lack of food. The urban apiary is located in the center of a medium city with heavy traffic (more than 110 thousand vehicles per day (GDDKiA 2020), on the rooftop of a 30-m high building. Therefore, bees are potentially exposed to different types of chemical pollutants from vehicles, higher temperatures because of the sun heated up the roof, and the presence of stronger air currents between buildings. Besides, bees use as a food base located in areas contaminated with many organic and inorganic pollutants. A summary of the general possible differences between urban and rural environment and general features of apiaries located in these areas based on the literature are presented, respectively, in Table 3 and Table 4.

**Table 3:** Several examples of important characteristics of urban environments referring to various aspects of environmental pollution and the degree of urbanization

Dominant features of urban areas compared to the rural areas	References
Higher population density, larger settlement size, higher patchiness and variation of landscapes, higher environmental burdens from fuel combustion, waste processing, water consumption (acid deposition, oxides emissions, waste and sewage production). Lower agricultural occupational profile.	McGranahan 2005
Higher total Cd, Cu, Ni, Pb and Zn concentration in soil (Aberdeen, United Kingdom).	Yang et al. 2006
Higher numbers of ozone days, the total number of days with fine particulate matter (PM 2.5), the better quality of the water quality (USA).	Strosnider et al. 2017
Higher level of nitrogen dioxide and sulfur dioxide (Maroussi municipality, Greece).	Priftis et al. 2007
Higher level of Cd, Cu, Hg, Pb and Zn, which mainly derived from anthropogenic inputs (Wuhan, China).	Gong et al. 2010
Higher level of local human wastes in the water, the domination of combustion fuel pollutants and specific to local production pollutants, more industries, more semi-natural landscapes like gardens, cemeteries, parklands (Hanoi, Vietnam; Los Angeles, USA).	McGranahan et al. 2004
Higher Pb, Zn, Cr, Ni, Sn, Cu, Mo, An concentration (Teresina, Brasil).	Mancarella et al. 2017

**Table 4:** Urban vs rural area—apiary features based on selected relevant literature (the most important)

Dominant features of apiaries	References
Urban areas	
Higher level of the contaminants in pollen, especially Al, Cd, Pb, and Co during July-October (southern-eastern Brasil).	Morgano et al. 2010
Indication of the markers of honey bees contamination: Al, As, and Cr on the bee body surface and Cd inside bodies. Slightly higher levels of As, Al, Pb, and Cd in bees (Warsaw, Poland).	Sadowska et al. 2019
Higher amounts of total phenols in honey (across Ireland)	Kavanagh et al. 2019
Metal (Pb, Cd, Cu, Fe, Zn, Mn, Ni, Cr, Hg), PAHs concentration in honey bee and pollen is within the regulatory level (Belgrade and vicinity, Serbia).	Jovetic et al. 2018
Higher deposits of particles (NO_3_^-^, SO_4_^2-^, NH4^+^) and gaseous pollutants (NO_2_, SO_2_ and NH_3_) and concentration of Al, Fe, Ti in pollen grains (Saitama, Japan).	Wang et al. 2009
Higher amounts of Pb, Cr, Cd in honeybee foragers (Abruzzi and Latium regions, Italy).	Perugini et al. 2011
More frequently measured the part of PAHs in honey bees registered during one year (5 of 20ies). 3-ringed substances were present often and more in autumnal than summer samples (14 departments, France).	Cochard et al. 2020
Rural areas	
Around 31 substances, the main rests were found in the case of amitraz, coumaphos, acrinathrin, cypermethrin, tau-fluvalinate as an effect of veterinary treatment and crop-contamination inside the hives (mainly beeswax or bee brood) (Córdoba, South Spain).	Morales et al. 2020
Higher level chlorpyriphos and acetamiprid in apiaries (across Spain).	Calatayud-Vernich et al. 2018
Contamination of honey with metals ( Cd, Mn, Cu) in dependence on local conditions and the use of pesticides or fertilizers in Umbria (35 cites, Italy)	Goretti et al. 2020
Higher risk of neonicotinoids and fungicides (UK).	David et al. 2016
Less number of microbial spores in honey (Belgrade and vicinity, Serbia).	Matović et al. 2018
Higher pesticides concentrations and more often detected presence in bumblebees from rural areas than from more urban areas (East Sussex, UK)	Botias et al. 2017

Our results revealed that the activity of GST and AChE did not differ significantly when taken into consideration any examined tissues in the bees from the urban and rural apiaries. It seems to be surprising these two parameters are important components of the stress response in invertebrates (e.g., Yan et al. 2013), and were proposed as a promising tool in the evaluation of honeybee health (e.g., Carvalho et al. 2013). Especially, changes in the AChE activities are presented as sensitive responses to pesticides that are widely used in agriculture also in the tested rural area (e.g., Nauen et al. 2001). Our results may indicate that bees from both apiaries were not seriously affected by pesticides, at least those from the neonicotinoid group. All the more so because artificial feeding of honey bees workers with different concentrations of Cd, Pb, and Cu in sucrose solutions changed GST and AChE activity and increased level of gene expression of the three classes of GST with increasing concentrations of Cu and Cd (Nikolić et al. 2019).

Analysis of the TAC revealed that the fat body was the only tissue that shows apiary localization-dependent differences. The higher TAC in the fat body of bees from the rural apiary was observed. There may be a need to elevate antioxidant protection against oxidative stress in bees from this localization. The varied prooxidant action of ingested food can be the possible source of differences in the antioxidant capacity between bees from the city and rural areas (Chainy et al. 2016; Słowińska et al. 2016). For example, the pollen and nectar that are collected by bees may differ in the content polyphenols and flavonoids, and thus the antioxidant capabilities (Aličić et al. 2014).

Our study revealed that foragers from the urban apiary differ in the level of the Hsp70 and defensins compared to the bees from the rural apiary. Besides, these responses are tissue-specific. These biomarkers indicate on processes of protection against stress factors among the others chaperone system toward proteins (Fink 1999; Kregel 2012; Saibil 2013) and activation of the immune system (Antúnez et al. 2009; Casteels-Josson et al. 1994).

The Hsp70 level was significantly higher in the thorax muscles, fat body, and gut of the foragers from the urban apiary than in the insects from the rural apiary. Hsps play the role of molecular chaperons of cells stabilizing proteins and inhibiting apoptosis. Their synthesis can also be induced by changes in ambient temperature as well as by toxins such as pesticides or metals (Fink 1999; Kregel 2012). So, enhancement of these proteins level may be connected with different reasons: a higher need to stabilize proteins in highly stressful conditions of surrounding urban apiary or may be connected with higher foraging activity.

The oxidative and mechanical damage that is induced by the intense work of the thorax muscles and the high temperatures that are generated by this work may be the primary reasons for the increased level of Hsp70 in this tissue. The strong heterothermia within individual foragers that had a thoracic temperature in a much higher level than normal (approximately 40 °C and higher) and the head temperature (10 to 14 °C) could be because the Hsp70 expression reduces any heat-induced damage (Elekonich 2009; Roberts and Harrison 1999). Furthermore, the location of the apiary on the rooftop of a high building (about 30 m) and the fragmentation of the food base could also be the main reasons for the intense muscle work (and consequently, cell-damage of its) in the bees from the urban apiary. It should be noted that a high level of Hsp70 may also be a manifestation of thermal tolerance in the thoracic muscles of the foragers from the urban apiary (Elekonich 2009).

The fat body of insects is the most metabolically active tissues and plays a crucial role in storing and utilizing energy and in detoxification processes. It is responsible for the synthesis of most of the hemolymph proteins (Arrese and Soulages 2010). The higher level of Hsp70 in the fat body of the foragers from the urban apiary might indicate a higher exposure of bees from the city to stress, but may also be a more appropriate response to any stress factors.

The gut is an organ that has direct contact with contaminated food, and thus as a target for various chemicals, it is extremely important from an ecotoxicological point of view as well as for insect studies (Malaspina and Mathias da Silva-Zacarin 2006). Numerous analyses have indicated that pollen can be contaminated with heavy metals (Conti and Botrè 2001; Lambert et al. 2012) or pesticides, even if it comes from wild plants (Long and Krupke 2016). Jabłoński et al. (1995) showed that there were almost three times more lead and six times more cadmium in the pollen than in the honey and in the nectar that had been collected directly from plants. These data indicate that foragers may be exposed to contaminants in high doses and over a long period of time. Our results revealed that the gut was the organ that had the highest Hsp70 level in the foragers from both the urban and rural apiaries. This may indicate that the guts of foragers from both apiaries could be the main route of xenobiotics action, and it seems the urban environment may be a bigger challenge in this case (e.g., connected with the pollution of nectar pollutants) than the rural environment because the Hsp70 level was higher in the guts of honey bees from the urban apiary.

The brain was the only organ in which no differences in the Hsp70 level were found. Similar results were reported by Elekonich (2009). It seems that the efficient defense mechanisms that protect the brain in other animals are an ancient evolutionary mechanism (Fink 1999) and it may also exist in honey bees. Protective role of transport barrier in the case of the central nervous system is valid in brain protection (Hindle et al. 2017). Moreover, it is possible important mechanisms of specific chemical defense, for example with cytochrome P450 system against neonicotinoid insecticides (Manjon et al. 2018).

The defensin level was slightly higher in the fat body and gut of the foragers from the urban apiary compared to the bees from the rural apiary. The fat body is the main site of the synthesis of the antimicrobial proteins that are synthesized under an injury and inductors (e.g., bacteria, fungi, and some insecticides) that may be present in the hemolymph (Ilyasov et al. 2012). The higher level of defensin in both the fat body and gut could indicate a possibility of existing of an infection in the foragers from the urban apiary. However, no symptoms of any disease were found. Moreover, the literature data indicate that the defensin level may be decreased by *Nosema* and *Varroa destructor* infection (Chaimanee et al. 2012). Furthermore, the *Lactobacillus*, which is non-pathogenic bacteria to bees, can stimulate the expression of defensin (Ilyasov et al. 2012).

To sum up, the increased level of the Hsp70 and defensins levels may mean that bees from the urban apiary are under greater pressure from stress factors in the city than in the rural area. This could mean that the multiple urban stressors may affect bees at both the individual and colony levels.

In our opinion, among selected biomarkers of urban honey bees’ multistress analysis, the most promising seems to be heat shock proteins and defensin, and secondly total antioxidant capacity. The most appropriate tissue for these investigations seems to be a fat body where the diverse answer for environmental conditions was observed at a statistically significant level. In addition, furthermore specific examinations are needed to show the main urban stressors which may pose a threat for honey bees’ populations in these promising apiary areas.

It would seem that the bees from the apiary located on the roof should have a greater muscle mass, which would enable them to fly to and from the apiary effectively. However, our results indicate that the mass of the muscles of the foragers from the urban apiary was significantly lower than the muscles of the foragers from the rural area. It is possible that, despite the difficult location of the hive and the fragmentation of the food base, the bees from the city do not have to fly over large distances like bees from rural areas. It is also possible that these foragers find food in close proximity to the apiary (e.g., in the parks inside the city or in flower beds) and that the warm air currents enable them to reach the roofs of buildings, and therefore, a high muscle mass is not necessary. However, Correa-Fernandez and Cruz-Landim (2010) revealed that the flight muscles from foraging workers might be resorbed and used as a supplementary source of energy. The reduction in thorax mass in flying insects is common, e.g., in nectar-feeding butterflies, but without affecting the flight performance negatively (Stjernholm et al. 2005). The reduction of muscle mass in bee foragers can be possible by damage due to flight wear down (Fernandez-Winckler and Cruz-Landim 2008). The high level of apoptosis-inhibiting proteins Hsp70 that was observed in our study in the thorax muscles in bees from the urban apiary can confirm these observations.

## Conclusions

Our study revealed that the multiple urban stressors may affect bees at both the individual and colony levels. In details, the level of two biomarkers of stress exposure (Hsp70 and defensins) differs among honey bee foragers from the urban and rural apiary. In addition, it seems that that the Hsp70 and the defensin levels and not the activity of GST and AChE are applicable biomarkers of stress exposure of the honey bees from apiaries that are located in the center of cities. To sum up, the welfare of honey bees from urban apiaries requires attention and more advanced further research.

Our conclusions from these studies are in contrast to those that were obtained by Badiou-Bénéteau et al. (2012, 2013) and Yan et al. (2013). However, it should be noted that they did not investigate bees from urban apiaries.

In light of the results that were obtained in this study, we begin work on creating a new experimental model. It consists of two professional apiaries (one located on the urban rooftop of the University building in Katowice, the center of the Silesian agglomeration and the other in rural areas—100 km from the center of the agglomeration) with genetically related honey bee colonies. In our opinion, such an experimental model will enable a much more reliable comparison between the welfare of honey bees from urban and rural apiaries and it will give the unusual possibility to compare various biological parameters of these colonies at different levels of their biological organization.

## Data Availability

The datasets used and/or analyzed during the current study are available from the corresponding author on reasonable request.
